# Sustainable mining of natural vein graphite via acid-extraction from waste attached to rock pieces of vein banks

**DOI:** 10.1038/s41598-023-42074-5

**Published:** 2023-09-07

**Authors:** Asiri D. T. Medagedara, Poornima Dahanayake, Herath Mudiyanselage T. G. A. Pitawala, Buddika Karunarathne, K. Kanishka H. De Silva, Masamichi Yoshimura, Kosala P. Walikannage, Thennakoon Mudiyanselage W. J. Bandara, Rajapakse Mudiyanselage G. Rajapakse, Gamaralalage R. A. Kumara

**Affiliations:** 1 National Institute of Fundamental Studies, Hantana Road, Kandy, 20000 Sri Lanka; 2 Postgraduate Institute of Science, University of Peradeniya, Peradeniya, 20400 Sri Lanka; 3 Department of Physics, University of Sri Jayewardenepura, Gangodawila, Nugegoda, 10250 Sri Lanka; 4 Graduate School of Engineering, Toyota Technological Institute, 2-12-1 Hisakata, Tempaku, Nagoya, 468-8511 Japan; 5 Central Environmental Authority, Rajapihilla Mawatha, Kurunegala, North Western Province Sri Lanka; 6 Department of Physics, University of Peradeniya, Peradeniya, 20400 Sri Lanka; 7 Department of Chemistry, University of Peradeniya, Peradeniya, 20400 Sri Lanka

**Keywords:** Materials science, Materials chemistry

## Abstract

A procedure based on acid extraction using a mixture of conc. sulfuric and nitric acids (8:1) to recover graphite attached to rock pieces of the vein contact zones of graphite mines, is developed as a sustainable mining practice. When the extracted graphite is heated at 600 °C for 15 min, it is converted to a highly expanded form resembling worm-like structures. The unique properties of this graphite and expanded graphite are presented by characterizing using FT-IR, Raman, SEM–EDX and XRD. This expanded graphite has the oil absorption capacity of 120 g of oil per 1 g of expanded graphite, making it the material so far known to have the highest oil absorption capacity. For comparison purpose, properties of ball-milled graphite powder which was obtained from the middle of the vein is prepared and characterized. However, the ball-milled graphite does not expand upon heat-treatment at 600 °C for 15 min. The acid-extracted graphite (AEG) has lower purity than that of ball-milled graphite (BMG), but heat-treatment increases the purity of the AEG while BMG shows opposite results. The purity of AEG has increased considerably upon heat-treatment by lowering the O wt% (weight percentage) by 6.07% to half of its original value while increasing C wt% by 8.05%. On the contrary, the C wt% of BMG has decreased by 3.71% and O wt% increased by 3.84%. The increase of purity upon heat treatment of AEG is due to the removal of some carbon and sulfur impurities as their volatile oxides. The ball-milled graphite absorbs carbon dioxide from the atmosphere when heat-treated at 600 °C. As such, the ball-milled graphite powder can be used to extract carbon dioxide from the atmosphere. The crystallite size of AEG is 1.25 times larger than that of BMG and it has been increased by 8 and 2.9 times, respectively, upon heat-treatment at 600 °C for 15 min. This is a clear evidence to expanded nature of AEG compared to BMG.

## Introduction

Graphite, historically known as black lead, is an industrial mineral with innumerable possible technological applications as a writing material, lubricant, refractory material, moderator in nuclear reactors, electrodes in power storage devices such as batteries and super-capacitors, electrodes for electrolyzers, and as a raw material for manufacturing expanded graphite and graphene products^1–5^. The estimated global reserves of graphite are 323.8 million tons where Turkey has the largest deposits followed by China and Brazil, which accounting to 73% of the world’s graphite reserves. The top ten graphite producing countries are China (200,000 MT), Brazil (68,000 MT), Mozambique (30,000 MT), Russia (27,000 MT), Madagascar (22,000 MT), Ukraine (17,000 MT), Norway (13,000 MT), Canada (8600 MT), India (65,000 MT) and Sri Lanka (4300 MT) totaling to almost 100% of graphite market, in the year 2021^6,7^. There are two types of naturally occurring economically important graphite: vein graphite, which is found in the veins of hard rocks, and flake graphite deposits, which occur in upper amphibolite to granulite facies metamorphic rocks as different types of ore deposits^8–10^. Ironically, only five countries in the world have vein graphite deposits and others are flake graphite deposits. The vein graphite deposits have been formed by the metamorphism of sedimentary rocks rich in carbonaceous matter or by the precipitation of carbon-bearing fluids (or melts) that formed inside the fractures of rocks^11^. Sri Lanka is the only country producing high-quality vein graphite to the global market^12^. The global graphite market size is projected to grow from $14.83 billion in 2021 to $25.70 billion in 2028 at a compound annual growth rate (CAGR) of 8.2% in the forecast period, 2021–2028^13^. The graphite market is expected to rise even further due to the rapid development of lithium ion and other batteries for electrical vehicles and due to increased high-tech applications of expanded graphite and graphene products^14–20^.

Exploiting vein graphite involves using explosives in pits and underground mines^21^. Graphite extraction is a cumbersome process that utilizes mechanical methods involving mining of graphite in chunk forms, hammering them to break into particles, powdering by ball-milling, and finally carbon enrichment by floating in water; the latter usually aided using froth formers, floating agents etc. The purity of graphite present in veins depends on the lateral dimensions of the vein; the purity is lowest at the walls of veins, and it gradually increases towards the middle at which the highest purity is obtained^22^. In Sri Lankan vein graphite, the purity of graphite mined from the middle of the vein exceeds 99% carbon that can be further purified to 99.99%. We have already developed a chemical-free flotation technology for graphite-enrichment^23^. The waste rocks of vein graphite mines contain considerable amounts of graphite attached to hard rock, since the usual mechanical methods of graphite extraction from those attached to hard rocks result in high contamination of gauge minerals and find rock particles. The mine waste rocks with attached graphite are piled up as waste in the graphite mining lands, and they may have an adverse effect on the environment^24^. Further, these rocks cannot be used in the construction industry due to the presence of graphite. In this research, we developed a novel and low-cost method to recover graphite attached to rock-pieces without contamination from impurities. Herein, we report the method we developed to recover graphite attached to pieces of rocks, and comparison of the graphite powder thus obtained with that produced by ball-milling of powdered graphite obtained away from the vein banks. This research is in line with sustainable development goals (SDGs) 8 (Decent Work and Economic Growth), 9 (Industry, Innovation, and Infrastructure), 12 (Responsible Consumption and Production) and 15 (Life on Land) where the SD is defined as development that meets the needs of the present generation without compromising the ability of future generations to meet their own needs. As such, the method that we developed is important in achieving these SDGs for recovering otherwise a waste to a useful material.

## Results and discussion

**Figure 1:**
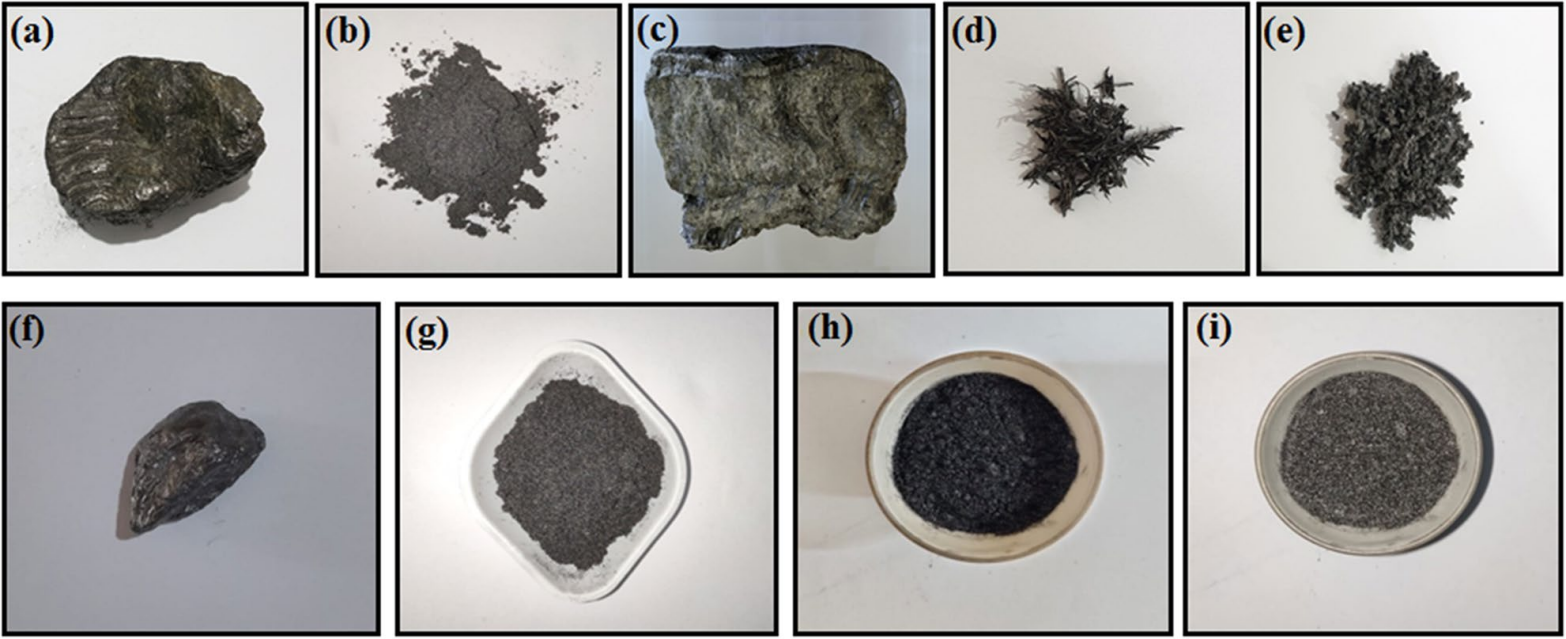
photographs of (**a**) a hard-rock piece with graphite attached on to its surface, (**b**) graphite powder formed by stirring graphite attached rock in the acid solution (AEG), (**c**) graphite removed rock, (**d**) AEG before heat-treatment, (**e**) AEG after heat-treatment, (**f**) graphite chunk obtained away from the vein wall without any rock fragments or mineral inclusions (**g**) BMG obtained from (**f**), (**h**) Ball-milled graphite (BMG) just before heat-treatment, and (**i**) BMG after heat-treatment.

Figure 1 shows the photographs of (a) a hard-rock with graphite attached on to its surface, (b) graphite powder formed by stirring graphite attached stone in the acid solution (AEG), (c) the rock after removal of graphite, (d) AEG before heat-treatment, (e) AEG after heat-treatment, (f) graphite chunk obtained away from the vein wall without any rock pieces (g) BMG obtained from (f), (h) BMG just before heat-treatment, and (i) BMG after heat-treatment. The comparison of the respective figures clearly shows that the acid-treatment gives less dense graphite particles, resembling morphology of worms, while ball-milling only reduces the particle size of the graphite.

The composition of the AEG and BMG, before and after heat treatment at 600 °C for 15 min., obtained from EDX data, are given in Table 1. Accordingly, the purity of AEG is low at 83.98% carbon wt% with considerable amounts of impurities such as O (12.47%), S (3.43%) and trace amounts of Al, Si and Fe. Interestingly, the heat treatment at 600 °C for 15 min has enriched the wt% of C by 8.05% with significantly reducing impurities to ~ half-their original values; O (6.64%), S (1.30%) (Table 1a). It seems that some of the C–O and S–O functionalities present in the untreated graphite have been removed as CO_2_ and SO_2_, due to heat-treatment, thus reducing the S and O wt%. Obviously, the BMG has a high C content of 97.55% with considerably lower impurity percentages as this graphite is obtained away from the vein walls. However, the heat treatment of this graphite at 600 °C for 15 min, has resulted in increase of O wt% by 3.85% and consequently decreasing C wt% by 3.71% possibly due to partial oxidation of ball-milled graphite. The results clearly distinguish two different trends on heat-treatment of the two types of graphite powders obtained.

**Table 1:** The composition of graphite obtained by (a) acid treatment of graphite attached rock piece (AEG) before and after heat treatment at 600 °C for 15 min. and (b) ball-milled graphite obtained from a graphite chunk (BMG) before and after heat treatment at 600 °C for 15 min.

Before heating at 600 °C for 15 min			After heating at 600 °C for 15 min		
Element	Wt%	$\pm \sigma$ Wt%	Element	Wt%	$\pm \sigma$ Wt%
(a)					
C	83.98	0.04	C	92.03	0.02
O	12.47	0.04	O	6.64	0.02
Al	0.03	0.00	Al	0.03	0.00
Si	0.05	0.00	S	1.30	0.00
S	3.42	0.01			
Fe	0.05	0.01			
(b)					
C	97.55	0.02	C	93.84	0.07
O	2.01	0.01	O	5.85	0.06
Al	0.06	0.00	Al	0.07	0.01
Si	0.19	0.00	Si	0.16	0.01
Ca	0.04	0.00	Fe	0.08	0.02
Fe	0.16	0.01			

**Figure 2:**
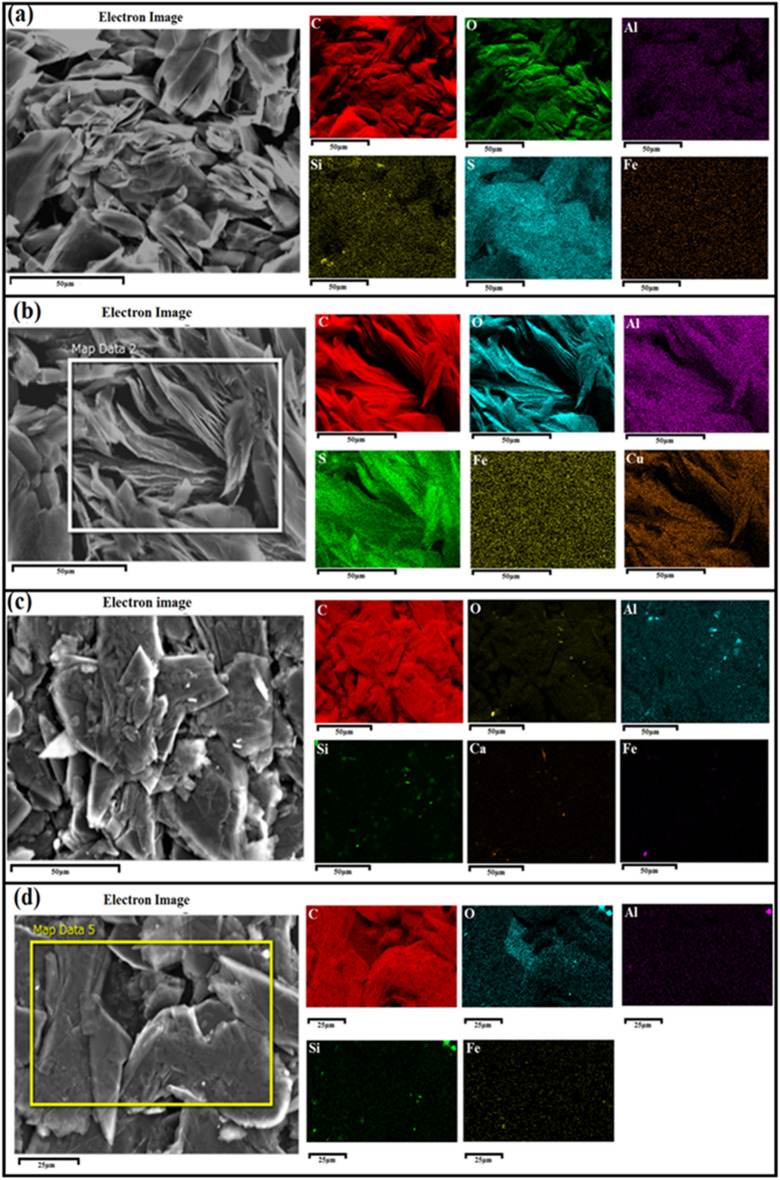
EDX data (**a**) before (scale—50 µm) and (**b**) after heat treatment (scale—50 µm) of acid-extracted graphite (AEG), (**c**) before (scale—50 µm) and (**d**) after heat treatment (scale—25 µm) of ball-milled graphite (BMG) together with map sum spectra and elemental wt%.

The EDX mapping of elements detected by the SEM–EDX (a) before and (b) after heat treatment of AEG and (c) before and (d) after heat-treatment of BMG together with map sum spectra and elemental wt% are shown in Fig. 2.

**Figure 3:**
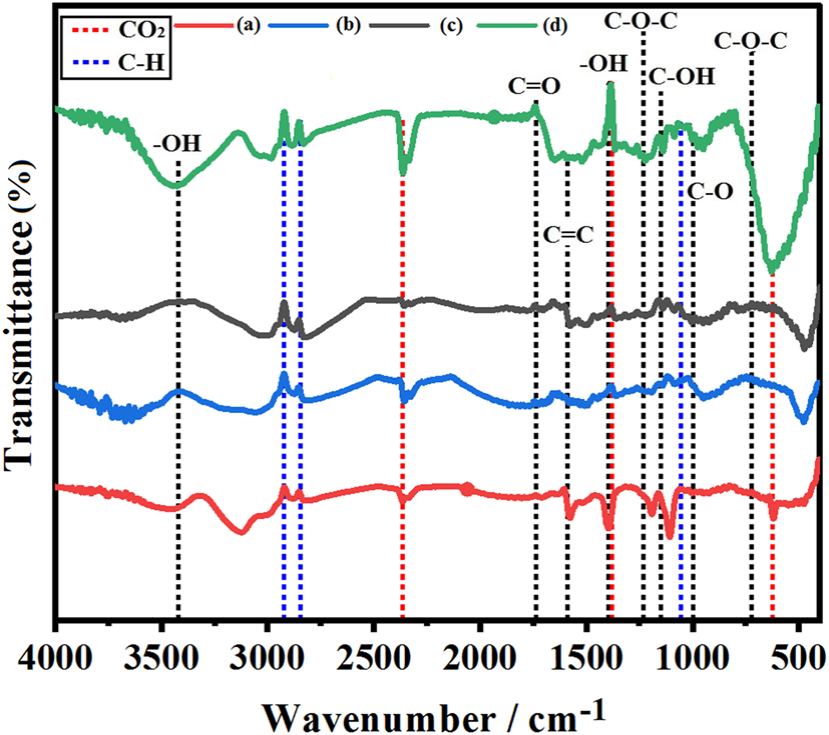
Labelled FT-IR spectra of acid-extracted graphite from those attached to rocks (**a**) before and (**b**) after heat-treatment, at 600 °C, for 15 min. and those of balled-milled graphite (**c**) before and (**d**) after heat-treatment.

Figure 3 shows the FTIR spectra of AEG (a) before and (b) after heat-treatment and BMG (c) before and (d) after heat- treatment. All the spectra contain the typical *sp*^2^ hybridized extended conjugated aromatic graphitic C=C bonds appearing at 1581 cm^−1^. The O–H vibration that is appearing at 3419 cm^−1^ is weak in AEG and is hardly observable in BMG without heat-treatment at 600 °C. Interestingly, this absorption band disappears in AEG due to heat-treatment but strongly appears in BMG after heat-treatment. The C–O vibration at 1000 cm^−1^ is clearly visible in AEG but only weakly appears in BMG before heat-treatment, at 600 °C. Again, this vibration band gets weakened in AEG, but its intensity increases considerably in BMG. The C-H vibration bands centered at 2923 cm^−1^ and 2845 cm^−1^ also become weaker in AEG but stronger in BMG upon heat-treatment. The C–O–H vibration at 1150 cm^−1^ is prominent in AEG without heat-treatment, but it gets weakened due to heat-treatment. For BMG, the trend of variation of the intensity of the C–O–H band is again opposite to that of AEG where it is weak before heat-treatment and gets stronger upon heat-treatment. The O–H deformation vibration band appearing at 1396 cm^−1^ also appears weakly in all four samples. The cyclic epoxy C–O–C vibrations appearing at 1235 cm^−1^ and 720 cm^−1^ are visible in AEG but are hardly visible in BMG. However, the heat-treatment reduces the intensity of these bands significantly in AEG but increases drastically in BMG. The C–H alkoxy vibration at 1054 cm^−1^ is also observable in all four samples. The C=O stretching vibration from carbonyl and carboxyl groups that is centered in 1738 cm^−1^ only weakly appears with almost no change due to heat-treatment in all four samples. All these results show that AEG has relatively higher amounts of oxygen functionalities than BMG without heat-treatment, at 600 °C. The heat-treatment at 600 °C for 15 min, drastically reduces the oxygen functionalities of AEG but significantly increases in BMG. The fact that higher amounts of oxygen functionalities are present in AEG when compared to those of BMG may be due to somewhat oxidation of the graphite structure by the conc. nitric and conc. sulfuric acids used in the extraction of graphite from those attached to rocks. Although ball-milling may introduces lattice defects (vide infra, Raman spectra) it is unlikely that it could lead to significant oxidation^25,26^. However, ball-milled graphite with more lattice defects can get oxygenated when heated at 600 °C in air. These results match exactly with those shown by the elemental analyses given in Table 1 where the purity of AEG has increased considerably upon heat-treatment by lowering the O% by 6.07% to half its original value while increasing C% by 8.05%. On the contrary, the C wt% of BMG has decreased by 3.71% and O% increased by 3.84%. Interestingly, the heat-treated BMG contains IR bands at 2362, 1388 and 667 cm^−1^ which can be assigned to asymmetric, symmetric, and doubly degenerated vibrations of carbon dioxide, respectively, but these bands are hardly seen in other samples even in the BMG without heat-treatment. This clearly shows that BMG absorbs carbon dioxide in air when heated at 600 °C but AEG does not.

**Figure 4:**
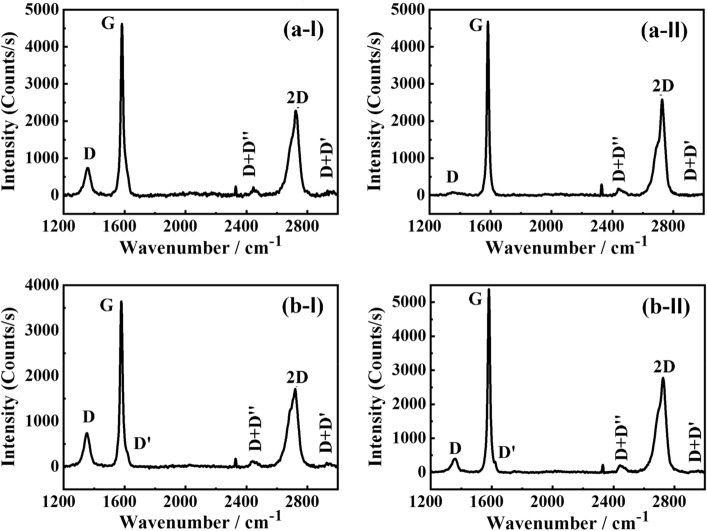
The Raman spectra of (**a**) acid-extracted graphite from those attached to rocks (a-I) before and (a-II) after heat-treatment and (**b**) ball-milled graphite (b-I) before and (b-II) after heat treatment.

Figure 4 shows the Raman spectra of (a) acid-extracted graphite from those attached to rocks (**a-I**) before and (**a-II**) after heat-treatment and (**b**) ball-milled graphite (**b-I**) before and (**b-II**) after heat treatment.

The Raman spectra of the AEG before and after heat-treatment, at 600 °C for 15 min, that are given in Fig. 4a-I and a-II show the following characteristics. The D-band characteristic to in-plane breathing mode vibration at the defect sites in sp^2^ hexagonal C rings appears at 1353 cm^−1^, the doubly degenerated G-band that is due to in-plane vibrations of *sp*^2^ hybridized conjugated carbon atoms, that appears at 1580 cm^−1^^27^. The intensity (I) of the D band is much lower than that of the G band with an I_D_/I_G_ ratio of 0.16 indicating a defect density n_D_ of 5.5 × 10^10^ cm^−2^ as calculated from Eq. (1) ^28^ before heat treatment. The heat-treatment of AEG at 600 °C for 15 min reduces the I_D_/I_G_ ratio to 0.02 after heat treatment suppress to a defect density 6.9 × 10^9^ cm^−2^. Thus, the defect density of AEG has decreased by a factor of 7.97 due to heat-treatment. This can be attributed to the removal of oxygen and sulfur functionalities due to heat treatment and thereby increasing the sp^2^ hybridized graphitic carbon sites. This agrees with the elemental analysis data presented in Table 1 where the impurity levels have been reduced by the same percentage when AEG is heat-treated at 600 °C for 15 min.

1 $$ {\text{n}}_{{\text{D}}} = { }\frac{{2.4{ } \times { }10^{22} }}{{{\uplambda }^{4} }}\frac{{{\text{I}}_{{\text{D}}} }}{{{\text{I}}_{{\text{G}}} }} $$

The D’ band that usually appears as a hump at the higher wavenumber side of the G band is hardly visible in AEG powder though the D + D″ band appear at 2440 cm^−1^. The 2D band appears at 2724 cm^−1^ with an I_2D_/I_G_ ratio of 0.49 before heat treatment. The heat-treatment increases the I_2D_/I_G_ ratio to 0.55 in AEG. The D + D’ band of AEG is also visible at 2930 cm^−1^.

The Raman bands of BMG before heat-treatment are as follows: at 1350 cm^−1^ (D band), 1578 cm^−1^ (G band), 2720 cm^−1^ (2D band), 2440 cm^−1^ (D + D’’) and 2927 cm^−1^ (D + D’) which deviate only slightly from the corresponding band positions of AEG. The I_D_/I_G_ ratio of BMG is 0.20 before heat-treatment which gives a defect density of 6.88 × 10^10^ cm^−2^. The I_2D_/I_G_ ratio of BMG before heat-treatment is 0.47. However, when BMG is heat-treated at 600 °Cfor 15 min, the intensity of the D band shifts to 1360 cm^−1^ (10 cm^−1^ hypsochromic shift) with a drastic reduction in its intensity thus giving rise to an I_D_/I_G_ ratio of 0.07 and a defect density of 2.41 × 10^10^ cm^−2^. The defect density of BMG has also decreased by a factor of 2.85. The peak position of the 2D band of BMG has shifted to 2725 cm^−1^ due to heat treatment yet, the I_2D_/I_G_ ratio has increased to 0.52. The decreased defect density is also evident from narrowing of the intensities of the XRD peak appearing at 2θ = 26.63° with a d-spacing of 3.35 Å in both types of graphite (vide infra). This shows that the heat-treatment of both AEG and BMG give almost stacking defect-free graphite with high crystallinity. The fact that 2D band is the second-most intense band of the Raman spectra suggests that the materials are highly ordered three-dimensional (3-D) and that decreased I_2D_/I_G_ ratio due to heat-treatment indicates increased 3D order^29^.

The crystallite size, ${\text{L}}_{{\text{a}}}$, was estimated using the following equation^30^.

2 $$ \frac{{{\text{L}}_{{\text{a}}} { }}}{{{\text{nm}}}} = { }\left( {2.4{ } \times 10^{ - 10} } \right){ }\left( {\frac{{\uplambda }}{{{\text{nm}}}}} \right)^{4} { }\frac{{{\text{I}}_{{\text{G}}} }}{{{\text{I}}_{{\text{D}}} }} $$

where ${\uplambda }$ is the wavelength of the laser used^30^. The calculated values of the crystallite size of AEG and BMG before and after heat-treatment are 104.7 nm, 837.6 nm, 83.8 nm, and 239.3 nm, respectively. This shows that the crystallite size of AEG is 1.25 times larger than that of BMG, before the heat treatment. The crystallite size of AEG and BMG have been increased by 8 and 2.9 times, respectively, upon heat-treatment at 600 °C for 15 min. These results show that the AEG is already present in an expanded form due to insertion of nitrate and sulphate groups within the interlayer spaces as predicted by the FT-IR data. Upon heat-treatment, AEG gets highly expanded, but BMG gets only moderately expanded. This is in accordance with their appearance before and after heat-treatment, as shown in Fig. 1.

**Figure 5:**
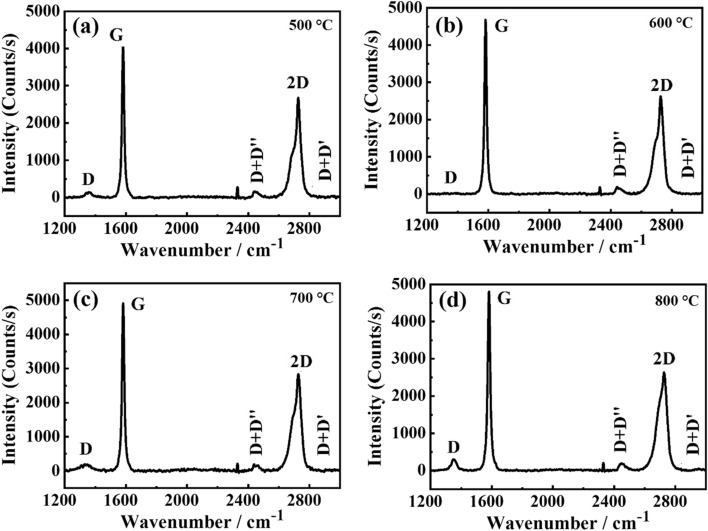
The Raman spectra of graphite obtained from those attached to rocks via acid extraction (AEG) after subjecting to heat-treatment at (**a**) 500 °C, (**b**) 600 °C, (**c**) 700 °C and (**d**) 800 °C.

Figure 5 shows the Raman spectra of AEG heat-treated at different temperatures. The intensity ratios of D and G bands and 2D and G bands are given in Table 2.

**Table 2:** The intensity ratio of D and G bands (I_D_/I_G_) and 2D and G bands (I_2D_/I_G_) for the graphite obtained from those attached to rocks via acid-extraction (AEG) after subjecting to heat treatment at different temperatures.

500 °C		600 °C		700 °C		800 °C	
I_D_/I_G_	I_2D_/I_G_	I_D_/I_G_	I_2D_/I_G_	I_D_/I_G_	I_2D_/I_G_	I_D_/I_G_	I_2D_/I_G_
0.04	0.65	0.02	0.55	0.04	0.58	0.06	0.55

Crystallite sizes of the expanded graphite obtained after heat-treatment of AEG at 500 °C, 600 °C, 700 °C, and 800 °C, for 15 min, are 418.8 nm, 837.6 nm, 418.6 nm, and 279.2 nm, respectively. As such, the largest crystallite size showing greatest expansion has been obtained at 600 °C. It is interesting to note that 600 °C is quite close to the crystallization temperature of graphite in the veins where it was extracted. Therefore, when AEG is subjected to heat-treatment at this temperature, the highest expansion is obtained.

**Figure 6:**
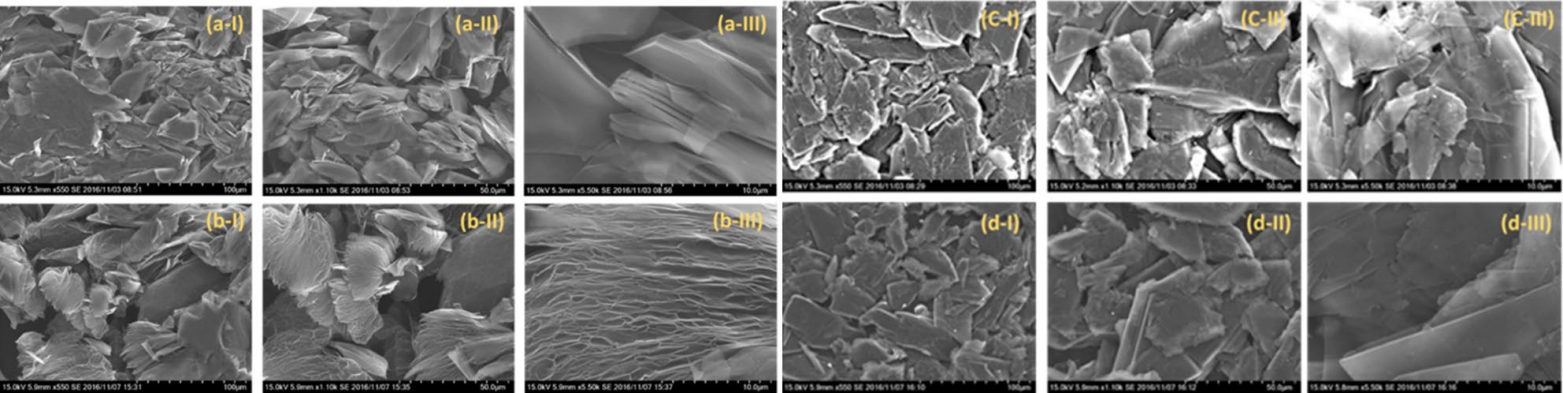
The SEM images of (**a**) graphite attached to rocks recovered from acid-treatment (AEG) before heat-treatment at (**a-I**) $\times$ 500 (a-II) $\times$ 1100 and (a-III) $\times$ 5500 magnifications. The corresponding SEM images of AEG after heat treatment are given in (**b**). The corresponding SEM images of (**c**) ball-milled graphite powder (BMG) before and after heat treatment, respectively, at different magnifications are given in (**c**) and (**d**).

Figure 6 shows the SEM images of (a) AEG before heat-treatment at (a-I) $\times \hspace{0.17em}$500 (a-II) $\times \hspace{0.17em}$1100 and (a-III) $\times \hspace{0.17em}$5500 magnifications. The corresponding SEM images of this graphite after heat treatment are given in (b). Figure 6c and d are the SEM images of BMG before and after heat treatment, respectively, at different magnifications. The SEM images of the two types of graphite powders are distinctly different showing different morphologies. The AEG has a kind of fine structure corresponding to particles with open thin sheets while the BMG has plate-like morphology. The expansion of the AEG is clearly visible as the distance between the graphite layers has been drastically increased due to heat-treatment as shown in (b-III) from that is shown in (a-III) before heat treatment. In BMG, only the plate-like morphology can be seen with somewhat expanded nature when c-III and d-III are compared. The heat-treatment has made AEG to be much less dense than that before heat-treatment due to significant increase in interlayer spacing as predicted by 8 times increased crystallite size (Raman spectroscopic data) but the increase in the interlayer spacing is moderate in BMG which are in good agreement with predictions made by the Raman spectroscopic data. Therefore, the acid-extraction method gives a convenient and straightforward two-step process for graphite extraction and expansion. The yield of the expanded graphite obtained by this method is over 95% but the yield is much less in BMG. The expanded graphite manufactured by the acid extraction from graphite attached to rocks has a unique structure resembling the morphology of worms. Interestingly, 1 g of the expanded AEG can absorb 120 g of petroleum oil and hence the material has a valuable application in oil spill cleaning and recovery^31^. The absorbed oil can be recovered simply by squeezing and the oil removed expanded graphite can be reused. We made a nylon mat containing this expanded graphite to clean large scale oil spills that usually occur during oil transportation in the sea. This is one of the major practical applications of the expanded AEG whereas the oil absorption capacity of expanded BMG is 2.7 times lesser than that of BMG. Interestingly, this ratio matches well with the ratio of expansion of AEG and BMG as obtained from Raman data. Therefore, it is clear that 8 times expanded AEG has a much higher oil absorption capacity than 2.7 times expanded BMG.

**Figure 7:**
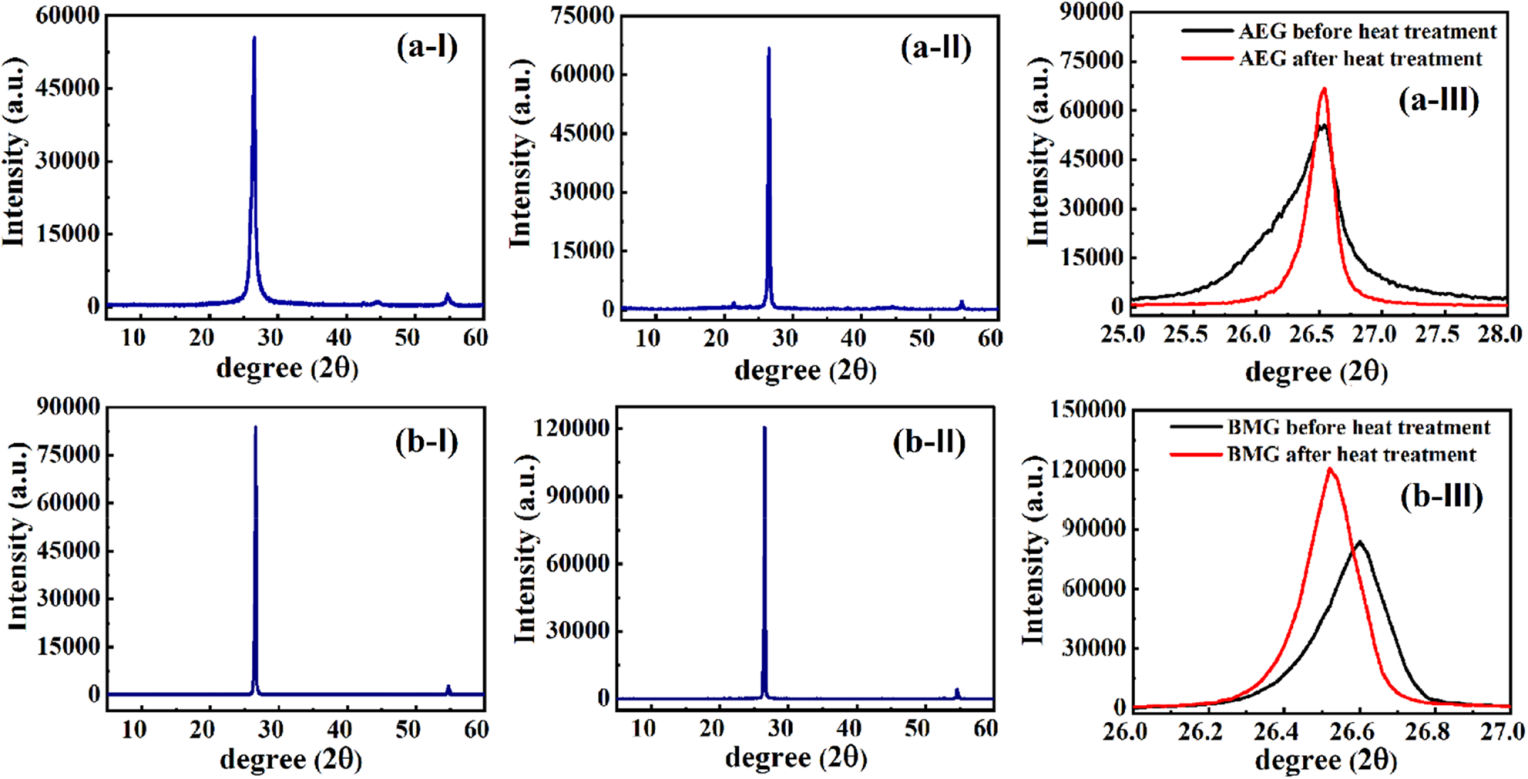
The X-ray diffractograms of (**a**) graphite attached to rocks that were subjected to acid treatment (**I**) before heat treatment (**II**) after heat treatment and (**III**) comparison of (**a-I**) and (**a-II**) at 2θ vales around the peak position and (**b**) balled-milled graphite powder (**I**) before subsequent heat treatment (**II**) after subsequent heat treatment and (**III**) comparison of (**b-I**) and (**b-II**) at 2θ vales around the peak position.

The X-ray diffractograms of AEG before and after heat treatment at 600 °C for 15 min. are shown in Fig. 7a-I and a-II, respectively, and those of BMG are given in Fig. 7b-I and b-II, respectively. The zoomed spectra before and after heat-treatment in the 2θ range from 25° to 28° for AEG and from 26° to 27° for BMG are shown in Fig. 7a-III and b-III, respectively.

Interestingly, the AEG has the dominant XRD peak centered at 2θ = 26.53° which corresponds to the diffraction from (002) plane and the interlayer spacing, d, calculated by applying the Bragg equation to this peak gives rise to 3.36 Å. The calculated values of the crystallite size along c axis, Lc, of AEG and BMG before and after heat-treatment are 11.5 nm, 32.9 nm, 39.8 nm, and 48.8 nm, respectively. This shows that the crystallite size, Lc, of AEG is 4.24 times smaller than that of BMG before the heat treatment. The crystallite size, Lc, of AEG and BMG have been increased by 2.86 and 1.23 times, respectively, upon heat-treatment at 600 °C for 15 min. This peak position does not change due to heat-treatment at 600 °C for 15 min, (Fig. 7a-I and a-II). However, the peak was broad before heat-treatment which appearing in the range from 25° to 28° with a full width at half-maximum (FWHM) of 0.7383°, but the heat-treatment has significantly sharpened reducing the FWHM to 0.25927° (Fig. 7a-III). The intensity of the peak has increased by 10,000 counts due to heat-treatment. This shows a significant decrease in the crystallinity of the AEG from 73.2 to 61.4% due to heat-treatment. In the BMG, the XRD peak corresponding to the diffraction from (002) plane appears at 2θ = 26.63° and it shifts to 26.53° upon heat-treatment (See Fig. 7 (b-III)) with a considerable increase in intensity of almost 40,000 counts. The FWHM has decreased from 0.21421° to 0.17483° upon heat-treatment. The decrease in 2θ corresponds to an increase of layer spacing by 0.01 Å. This result is consistent with the composition data obtained where the graphite obtained from acid-treatment of those attached to rocks of vein walls show increased purity due to heat-treatment, but the ball-milled graphite gets increased O wt%. It is, therefore, possible that the former contains in-plane O- and S-functional groups and some of which are removed by heat-treatment without changing the interlayer space of 3.36 Å. However, the latter gets oxidized to some extent and the O-functional groups such as epoxy bridges are introduced into the interlayer space thus increasing the interlayer spacing by 0.01 Å.

## Conclusion

A two-step process involving acid extraction followed by heat-treatment at 600 °C for 15 min results in the extraction and expansion of graphite attached to rock pieces of the vein banks of graphite mines. This novel and low-cost method introduced to recover the graphite attached to rock pieces which in general wasted during the graphite extraction process is helpful to reduce the impurities. The purity of this graphite increases due to heat-treatment as a result of elimination of carbon and sulfur as their volatile oxides. The expanded graphite thus formed has a worm-like morphology and absorbs 120 g of spilled oil per 1 g of expanded graphite. The graphite obtained away from the vein bank and ball-milled to reduce particle sizes has a higher purity than those obtained from the vein bank but the purity of the former decreases upon heat-treatment due to absorption of carbon dioxide from air. As such, the ball-milled graphite can be used to directly capture carbon dioxide from air.

## Methods

### Refining graphite attached to the wall rock

The rock pieces with attached graphite on them were dipped in a solution containing conc. H_2_SO_4_ and conc. HNO_3_ in 8:1 v/v ratio, and lightly shaken with a glass rod. The size of the rock pieces is immaterial as long as they can be immersed in the acid solution. After a few minutes, delamination of graphite pieces was observed, and graphite particles thus formed are dispersed and some are settled in the solution. The rocks obtained after delaminating graphite attached to them were removed from the solution. The solution containing both dispersed and settled graphite particles was then filtered and the filtrate was reused to extract more graphite from rocks containing attached graphite. The graphite particles present in the residue were washed several times with distilled water until the effluent became neutral. This graphite is named acid-extracted graphite (AEG). For comparison purpose, graphite obtained from the middle of the vein without contamination was ball-milled (Ball milling parameters, Ball-to-powder ratio (BPR) = 5:1, milling media of bowls and balls made of hardening stainless steel, bowl volume of 250 ml, ball diameter of 10 mm, milling time of 2 h, milling speed of 500 rpm, and each ball weighing approximately 4.2 g) and powdered to prepare ball-milled graphite (BMG). Photographs of samples were taken at different stages.

### Characterization

Both the AEG and BMG were characterized by Fourier-transformed infrared spectroscopy (FT-IR, Bruker Alpha FTIR spectrometer in the range 500–4000 cm^−1^), X-ray diffraction (XRD, Rigaku, (RINT-TTR III), Cu Kα radiation (λ = 1.5406 Å), Scanning electron microscopy coupled with energy dispersive X-ray analysis (SEM–EDX, FE-SEM, Hitachi S-4700), and Raman spectroscopy (Renishaw Invia Reflex Raman microscopy system). Both the AEG and BMG were separately heated at 600 °C for 15 min in a box furnace and the heated products were also characterized by the above methods. The FT-IR and Raman spectra were recorded after heat-treating at 150 °C for 2 h to remove any adsorbed moisture present in graphite samples. The FT-IR was done by making a pellet using a pellet press (Specac, Manual Hydraulic Press 15 Ton GS15011) with dried graphite and dry KBr mixed in 1:20 V/V ratio. Raman spectra were obtained by individually heat-treating AEG samples at 500 °C, 600 °C, 700 °C, and 800 °C for 15 min each, aiming to identify the optimum temperature for the heat treatment.

## Data Availability

All data generated or analyzed during this study are included in this published article.
